# Inverse Kinematics for Upper Limb Compound Movement Estimation in Exoskeleton-Assisted Rehabilitation

**DOI:** 10.1155/2016/2581924

**Published:** 2016-06-15

**Authors:** Camilo Cortés, Ana de los Reyes-Guzmán, Davide Scorza, Álvaro Bertelsen, Eduardo Carrasco, Ángel Gil-Agudo, Oscar Ruiz-Salguero, Julián Flórez

**Affiliations:** ^1^eHealth and Biomedical Applications, Vicomtech-IK4, Mikeletegi Pasealekua 57, 20009 San Sebastián, Spain; ^2^Laboratorio de CAD CAM CAE, Universidad EAFIT, Carrera 49 No. 7 Sur-50, 050022 Medellín, Colombia; ^3^Biomechanics and Technical Aids Department, National Hospital for Spinal Cord Injury, SESCAM, Finca La Peraleda s/n, 45071 Toledo, Spain

## Abstract

Robot-Assisted Rehabilitation (RAR) is relevant for treating patients affected by nervous system injuries (e.g., stroke and spinal cord injury). The accurate estimation of the joint angles of the patient limbs in RAR is critical to assess the patient improvement. The economical prevalent method to estimate the patient posture in Exoskeleton-based RAR is to approximate the limb joint angles with the ones of the Exoskeleton. This approximation is rough since their kinematic structures differ. Motion capture systems (MOCAPs) can improve the estimations, at the expenses of a considerable overload of the therapy setup. Alternatively, the Extended Inverse Kinematics Posture Estimation (EIKPE) computational method models the limb and Exoskeleton as differing parallel kinematic chains. EIKPE has been tested with single DOF movements of the wrist and elbow joints. This paper presents the assessment of EIKPE with elbow-shoulder compound movements (i.e., object prehension). Ground-truth for estimation assessment is obtained from an optical MOCAP (not intended for the treatment stage). The assessment shows EIKPE rendering a good numerical approximation of the actual posture during the compound movement execution, especially for the shoulder joint angles. This work opens the horizon for clinical studies with patient groups, Exoskeleton models, and movements types.

## 1. Introduction

Robot-Assisted Rehabilitation (RAR) supplements conventional therapy in the treatment of nervous system injuries (e.g., stroke and spinal cord injury), as robots enable repetitive, task-specific, intensive, and interactive treatment [1–3]. In RAR, the accurate estimation of the patient limb posture (i.e., determination of joint angles) is a fundamental prerequisite for the following.

1. The verification of the compliance of the patient movements with the prescribed exercises: patient movements must follow the medically prescribed ones, without using the healthy joints to compensate for treated joints [4, 5].
2. The long-term assessment of the patient evolution: objective evaluation methods based on the analysis of the patient kinematic data have been recently developed [6–8] to overcome the limitations (subjectivity, low sensitivity [9]) of traditional scales (e.g., Barthel Index [10], Functional Independence Measure [11]) to assess the functionality of a patient.

Traditional motion capture systems (MOCAPs), such as optical, electromagnetic, and inertial ones, have been used in many rehabilitation scenarios to accurately estimate the human posture [12–14]. However, the use of the currently existing MOCAPs in Exoskeleton-based RAR is impractical because the Exoskeleton body causes optical occlusions and magnetic disturbances in the MOCAP components. Furthermore, in RAR therapies involving functional electrical stimulation (e.g., [15]) and/or electromyography the markers or sensors of the MOCAP interfere with the setup. Even if MOCAP devices can be arranged to coexist with the Exoskeleton, the operation is complex and incompatible with the time and resources available for a typical patient appointment. Therefore, they can be used in specific assessment sessions but not for daily patient attention.

In Exoskeleton-based therapy, the prevalent approach to estimate the limb joint angles is to approximate them directly with the Exoskeleton joint angles (e.g., [8, 16–18]). However, the accuracy of this strategy is limited by the differences between the kinematic structure of the patient limb and Exoskeleton [6]. In the case of the upper limb (focus of this research), a direct accurate measurement of the shoulder angles is particularly difficult, since it demands an Exoskeleton with a complex kinematic model that considers the simultaneous motion of the sternoclavicular and acromioclavicular joints.

Computational methods in [19–21] for Exoskeleton-based therapy estimate the arm swivel angle, which parametrizes the arm pose [22]. Let *W* be a plane defined by the central points of the Gleno-Humeral (GH), elbow, and wrist joints. Then, the rotation angle of the plane *W* around the axis that goes from the wrist to the GH joint is defined as the swivel angle [19]. In these methods, the Inverse Kinematics (IK) of the arm (which is redundant) is solved by estimating a swivel angle that allows the subject to retract the palm to the head efficiently. Results in [19, 20] are improved in [21] by considering the effect of the wrist orientation on the swivel angle estimation. These references do not report how the error in the swivel angle estimation is traced to individual errors in the wrist, elbow, and Gleno-Humeral (GH) joint angles.

The method in [21] requires (a) the position of the GH joint center, (b) the pose of the wrist, (c) the initial position of the elbow, and (d) a point in the head neighborhood that minimizes the swivel angle estimation error. In typical clinical scenarios, the mentioned inputs are unavailable. This circumstance makes the method in [21] difficult to apply. For extended discussion, see [22, 23].

Acknowledging different kinematic structures in limb and Exoskeleton, [23] introduces the EIKPE (Extended Inverse Kinematics Posture Estimation) method. EIKPE considers the parallel kinematic chains limb and Exoskeleton as related through the cuff constraints that fix them together. EIKPE then solves the IK problem of the parallel chain, therefore finding the limb joint angles. The real-time EIKPE accuracy (circa 3-degree RMS) is reported for (1) wrist flexion-extension, (2) elbow flexion-extension, and (3) forearm pronation-supination. Limitations of [23] are (a) restriction to 1-DOF movements due to constraints in the ground-truth reading equipment and (b) elbow and wrist angle estimations.

*Contributions of This Paper.* The present paper complements [23] (see Table 1) by addressing the training of compound movements (simultaneous movement of multiple joints). In particular, it is shown how EIKPE enhances the accuracy in the estimation of the GH joint angles with respect to the Exoskeleton-based approach. Specifically, contributions of this paper are as follows.

1. The paper illustrates the capacity of EIKPE in addressing compound movements (i.e., object prehension), extending the results of [23], which had individual joint movements. This added complexity requires the usage of (a) more evolved marker and camera sets, (b) a more complex biomechanical and kinematic model, and (c) an optimized posture estimation for full arms.
2. It computes the error in the GH and elbow joint angles of EIKPE with respect to the measurements of a marker-based optical MOCAP.
3. It computes the error in the GH and elbow joint angles of the rehabilitation Exoskeleton encoders with respect to the measurements of the MOCAP.
4. It applies various statistical measures (linear fit method (LFM), RMSE, ROM error, box plots, and significance test) to assess the differences between items (3) and (4), showing the feasibility of using EIKPE to enhance posture estimates from Exoskeletons.

Table 1 shows further details on the differences and contributions of the present paper when contrasted with related publications.

## 2. Materials and Methods

This section briefly introduces EIKPE and describes how the ground-truth values are used to assess the accuracy of the angle estimations provided by EIKPE and Exoskeleton joints.

### 2.1. EIKPE Method

Since the purpose of the present paper is the experimental assessment of the theoretical construct in [23], only the key aspects of EIKPE are discussed here.

**Figure 1:**
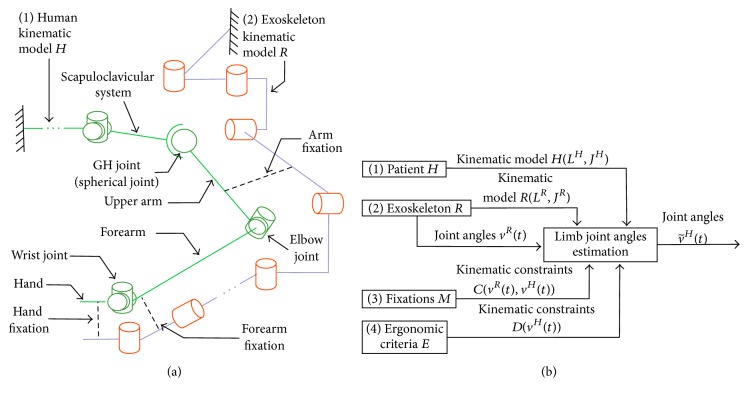
(a) Schematic diagram of the human and Exoskeleton kinematic models and their interaction. (b) Inputs and outputs of the limb posture estimation algorithm.

To estimate the angles of the limb joints of the patient (denoted by vector *v* ^*H*^(*t*)) during RAR, the human limb and Exoskeleton are modeled as a parallel kinematic chain connected by the fixations of the Exoskeleton (Figure 1(a)).

The elements that are considered inputs to the problem are as follows(Figure 1(b)).

*(1) Patient.* The human limb kinematic model is denoted by *H*(*L* ^*H*^, *J* ^*H*^) (e.g., the Denavit-Hartenberg parameters [24]), where *L* ^*H*^ and *J* ^*H*^ are sets of links and joints, respectively. The human kinematic model used in EIKPE includes joints of the spine, scapuloclavicular system, and arm. The upper limb is modeled with 9 DOFs: 2 DOFs of the scapuloclavicular system, 3 DOFs of the GH joint (spherical joint), 2 DOFs of the elbow, and 2 DOFs of the wrist.

*(2) Exoskeleton.* The Exoskeleton kinematic model is denoted by *R*(*L* ^*R*^, *J* ^*R*^). The Exoskeleton joint angles are denoted by vector *v* ^*R*^. The values of *v* ^*R*^ at any instant *t* of the therapy (*v* ^*R*^(*t*)) are known. In the rehabilitation platform where EIKPE is implemented the Exoskeleton corresponds to the Armeo Spring® (Hocoma, AG) [25], which has 7 DOFs.

*(3) Set of Fixations M*. The fixations *M* are passive mechanisms that connect the Exoskeleton and the patient. *C*(*v* ^*H*^(*t*), *v* ^*R*^(*t*)) is the set of vector-valued functions that model the kinematic constraints imposed by the fixations *M* to the patient limb.

*(4) Set of Ergonomic Criteria E*. *E* consists of a set of principles that dominate the posture of the patient limb while interacting with the Exoskeleton (e.g., the preference of the human to put the limb in a rest posture *v*_rest_^*H*^). *D*(*v* ^*H*^(*t*)) is the set of vector-valued functions that model the kinematic constraints imposed on the patient limb by the set of ergonomic criteria *E*.

The goal of the implemented algorithm is to find the approximate angles of the joints of the patient limb ${\tilde{v}}^{H}(t)$, such that the sets of constraints *C* and *D* are met.

In order to obtain the estimations ${\tilde{v}}^{H}(t)$, the IK of *H*(*L* ^*H*^, *J* ^*H*^) is solved considering the sets of constraints *C* and *D*. The IK solution is obtained in real-time using the V-REP® simulator (Coppelia Robotics, GmbH) [26]. The joint angles of the Exoskeleton and EIKPE are sampled with frequency *f*_*s*_ = 60 Hz.

### 2.2. Ground-Truth Motion Capture and Analysis

#### 2.2.1. Biomechanical Model

**Figure 2:**
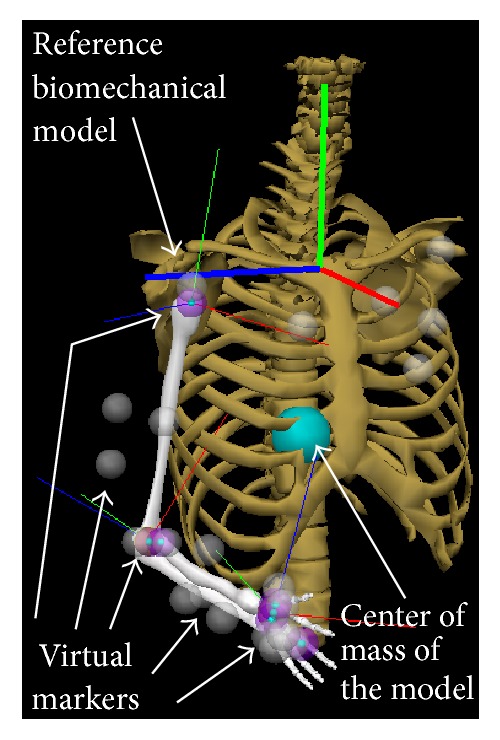
Reference biomechanical model and virtual markers for motion reconstruction [27].

The biomechanical model (Figure 2) of the upper limb described in [27] was used as the reference kinematic model for the assessment of the accuracy of EIKPE. This model was developed in the software Visual3D*™* (C-Motion, Inc.) [28] and presents 6 DOFs: 3 DOFs of the GH joint (spherical joint), 2 DOFs of the elbow joint, and 1 DOF of the wrist joint. The biomechanical model can be scaled to match the anthropomorphic measures of each of the test subjects.

The biomechanical model includes virtual markers (gray spheres) that allow reconstructing the motion of the limb by using motion data from MOCAPs. In order to do so, the 3D positions of the real markers (which were installed on the patient and tracked by a MOCAP) are treated as the desired positions of the virtual markers. Then, the limb joint angles are computed by solving the IK of the limb such that the position of the virtual markers matches the position of the real markers. The detailed geometry depicted in Figure 2 is only used for visualization purposes and a simplified version is used in the IK computation. The joint angles obtained by using this methodology are the ground-truth *v* ^*H*^(*t*) angles.

#### 2.2.2. Marker Placement Protocol

**Figure 3:**
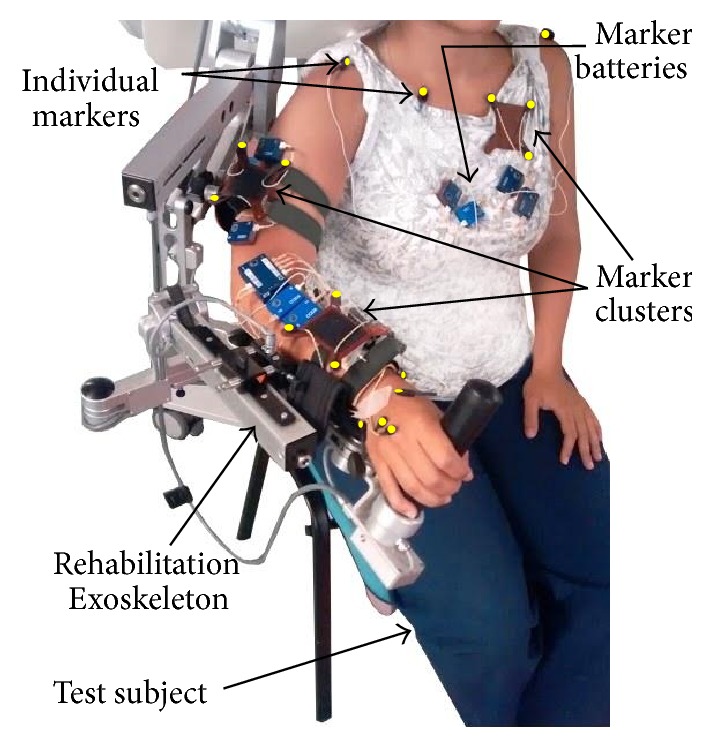
Setup of the markers (highlighted in yellow) of the MOCAP system.

A total of 21 markers are installed on each test subject to precisely track the movement of the upper limb. The markers are distributed on the subject arm and trunk as described in Table 2 and Figure 3.

#### 2.2.3. Motion Capture System

**Figure 4:**
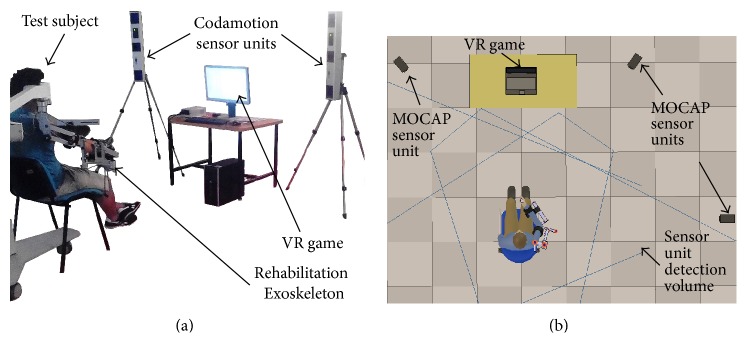
Setup for motion capturing in RAR: (a) MOCAP sensor units and (b) their distribution around the test subject.

The Codamotion® (Charnwood Dynamics Ltd.) [29] is an optical marker-based MOCAP. This MOCAP uses active markers that emit infrared light, which is detected by 3 sensor units (Figure 4(a)). For the accuracy assessment experiments, the MOCAP sensor units are distributed as depicted in Figure 4(b). With the described marker setup, the marker position sampling frequency is *f*_*c*_ = 200 Hz.

#### 2.2.4. Experimental Protocol

**Figure 5:**
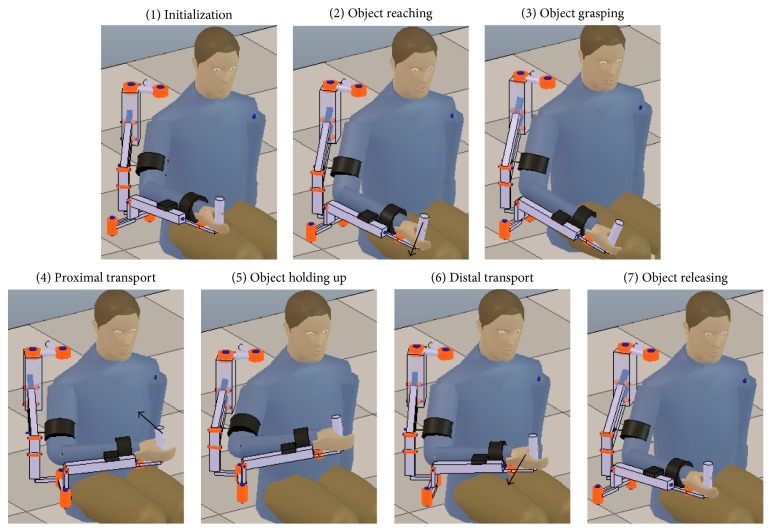
Stages of the prehension ADL. Black arrows indicate the approximate direction of movement of the hand of the test subject.

The functional task that was chosen to conduct the accuracy assessment is the activity of daily living (ADL) of prehension, which has its stages shown in Figure 5. Notice that the prehension task shares movement stages with other ADLs, such as drinking and eating, which are among the most relevant tasks to rehabilitate [30].

The prehension movements are performed with the forearm pronation-supination and the wrist flexion-extension DOFs blocked in the Exoskeleton in order to avoid marker occlusions during the ADL movement (such joint blockage does not affect the angle estimation capabilities of the MOCAP or EIKPE). The joint angles of the blocked DOFs are not studied in this work.

In the setup stage of this protocol, the lengths of the arm and forearm of each test subject are manually measured and entered into the EIKPE software (as it would be done in a clinical application). The Exoskeleton arm and forearm link lengths are adjusted for every subject according to the device manufacturer instructions. The Exoskeleton link lengths are also entered into the EIKPE software. Next, the optical markers are installed on the subject and the MOCAP calibration procedure is conducted.

After the subjects wear the Exoskeleton, they perform some practice trials with the virtual reality (VR) game. In the VR game, the hand positions at the grasping and object holding up stages are calibrated for each subject. For each test subject, 4 repetitions of the prehension movement are recorded. Each prehension movement execution is limited to 20 seconds. A total of 4 healthy subjects participate in the movement recordings.

#### 2.2.5. Signal Processing and Analysis

The accuracy assessment presented in this paper involves the comparison of the upper limb joint angle estimates that come from the following sources:

- The joint angles obtained from the MOCAP.
- The joint angles obtained from EIKPE.
- The joint angles obtained from the Exoskeleton encoders.

Table 3 summarizes the measured angles of the joints of the upper limb, the methods and reference coordinate systems (CS) used to compute such joint angles.

In order to compare the various joint angle measurements along the execution of the prehension movement, the obtained joint angle signals are filtered and synchronized as follows.

*(1) Resampling and Filtering.* The joint angle profiles obtained from the MOCAP are resampled to match the sampling frequency of the Exoskeleton and EIKPE. Then, a low-pass Butterworth filter with a 5 Hz cutoff frequency is applied to all the obtained signals. Figures 7(a)–7(c) show the angle estimations of the elbow flexion of one of the trials of a subject after resampling and filtering.

*(2) Signal Trimming.* The joint angle profiles obtained from EIKPE and Exoskeleton are manually trimmed such that they approximately contain the same movement segment recorded with the MOCAP.  Figure 7(d) shows the trimmed Exoskeleton and EIKPE estimations of the movement trial mentioned in the previous step.

*(3) Signal Reference Adjustment.* The coordinate systems of reference of the MOCAP, EIKPE, and Exoskeleton are not registered to each other, which impedes transforming the angle estimations to a common coordinate system to compare them. In order to compare the angle estimations, they are related to each other by using the steady limb joint angles at the initialization posture (subjects were asked to remain static in this posture for a few seconds). To do so, the joint angles measured by the MOCAP at the initial stage of the movement are set as the initial values for the angle estimations of the Exoskeleton and EIKPE. In this way, the estimations of the joint movements performed with respect to the initialization posture can be compared.  Figure 7(e) shows an example of the result of this step.

*(4) Temporal Axis Offset Adjustment.* A fine tuning in the aliment of the signals in the temporal axis is performed by applying a time offset to the EIKPE and Exoskeleton estimations such that their correlation with the MOCAP measurements is maximized.  Figure 7(f) shows an example of the result of this step.

After synchronization of the joint angle signals, the following error metrics are computed.

*(1) Error in the Estimation of the ROM.* The amplitude of the Exoskeleton and EIKPE joint angles are compared with the ones of the MOCAP.

*(2) RMS Error (RMSE) of the Joint Angle Profiles.* The RMS of the pairwise differences between the joint angle profiles of the Exoskeleton and EIKPE with respect to the ones of the MOCAP are computed.

*(3) LFM Parameters.* The LFM [31] is applied to compare the waveforms of the reference *D*_ref_ ∈ *R* ^1×*N*^(MOCAP) and estimated angles *D*_est_ ∈ *R* ^1×*N*^ in terms of the linear regression coefficients: *A*: offset, *B*: amplitude, and *R* ^2^: shape similarity [32]. These coefficients are computed such that *D*_fit_ = *A* + *B∗D*_ref_ minimizes ∑_*j*=1_^*N*^(*D*_est__*j*_ − *D*_fit__*j*_)^2^. Notice that if *D*_est_ = *D*_ref_ (ideal fit), the LFM coefficients take the following values: *A* = 0, *B* = 1, and *R* ^2^ = 1.

The obtained ROM error and RMSE metrics of the Exoskeleton and EIKPE are compared with a paired difference test to check if there is a statistically significant difference between their means (confidence interval 95%).

## 3. Results and Discussion

Table 4 presents the average RMSE and ROM errors (± their standard deviation) of the joint angles measured by the Exoskeleton and EIKPE for all the trials of the test subjects when compared to the joint angles provided by the MOCAP (ground-truth). Around 12200 samples were compared to compute each of the RMSE values presented in Table 4. A Wilcoxon signed-rank test [33] was performed to check if there is a statistically significant difference between the mean accuracy of the methods in estimating the various joint angles and ROMs (by using the SPSS statistical analysis software (IBM Corp.) [34]). Values in bold in Table 4 indicate statistically significant differences between the accuracy provided by the Exoskeleton and EIKPE.

Table 5 presents the assessment of the estimation methods according to the LFM. In this table the average and standard deviation of the parameters are presented for each studied angle.  Figure 8 shows the application of the LFM to one of the SAA datasets. When the waveforms of the estimated and reference (MOCAP) angles (Figure 8(a)) are similar, a linear fit of the angle estimations (black dashed line in Figures 8(b) and 8(c)) that resembles the ideal linear fit (blue line, *A* = 0, *B* = 1, and *R* ^2^ = 1) is obtained. The ideal linear fit represents the case of a perfect match between the reference and estimated angles.

For the case in Figure 8, EIKPE estimations closely approximate those of the MOCAP, and the LFM parameters are close to those of the ideal linear fit (blue and black lines are close to each other in Figure 8(c)). The waveform of the Exoskeleton estimations presents similar shape (*R* ^2^ ≈ 1) but different amplitude (*A* ≪ 0 and *B* ≫ 1) compared to those of the MOCAP. The effects of the values of parameters *A* and *B* are reflected in the offset and slope of the linear fit of the Exoskeleton estimations (Figure 8(b)), which poorly approximates the ideal linear fit.

### 3.1. Angle Estimations of the GH Joint

The RMSE, ROM, and LFM metrics (Tables 4 and 5) show that EIKPE presents small errors in estimating the SFE and SAA angles. In comparison with the results obtained for SFE and SAA, EIKPE presents larger errors in the SIER angle estimation. EIKPE estimates the SFE and SAA angles using the movement constraints imposed by the Exoskeleton on the upper arm. However, the estimation of the SIER angle involves information of the pose of the forearm (which also depends on the elbow movement) and therefore is subject to additional estimation and modeling errors. In Figure 9, it can be observed that EIKPE angle waveforms follow closely those of the MOCAP. For the GH joint angles, there is a strong correlation (0.82 ≤ *R* ^2^ ≤ 0.91) between EIKPE and MOCAP estimations.

**Figure 6:**
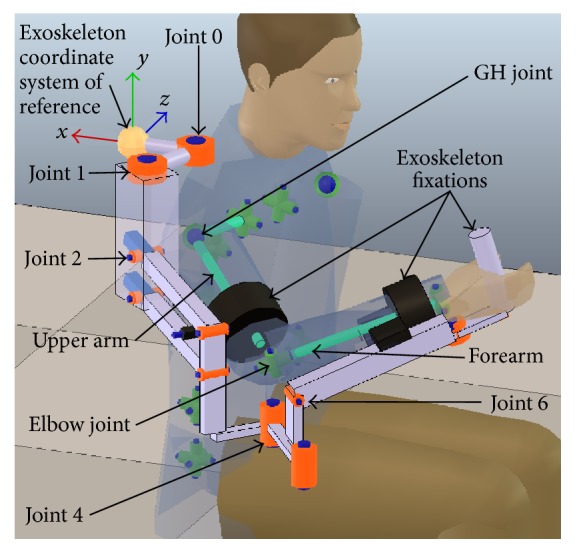
Human and Exoskeleton kinematic models and their joints of interest for the experiments.

**Figure 7:**
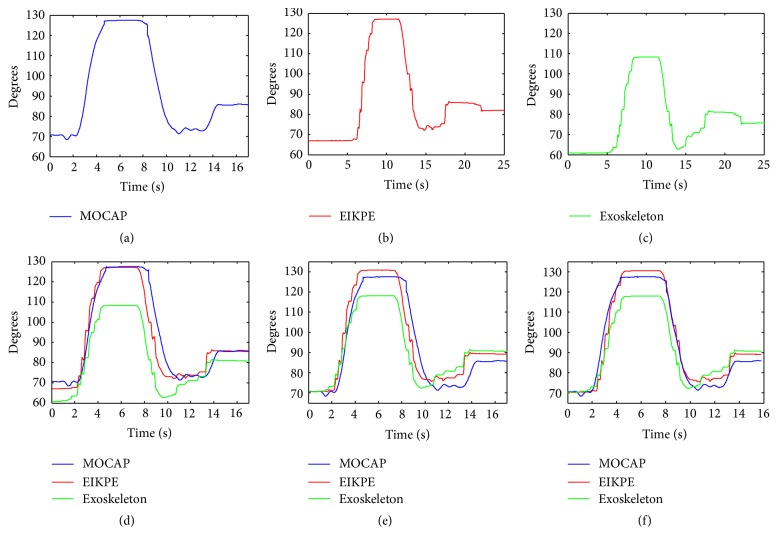
Signal synchronization process of elbow flexion angle estimations from a movement trial.

**Figure 8:**
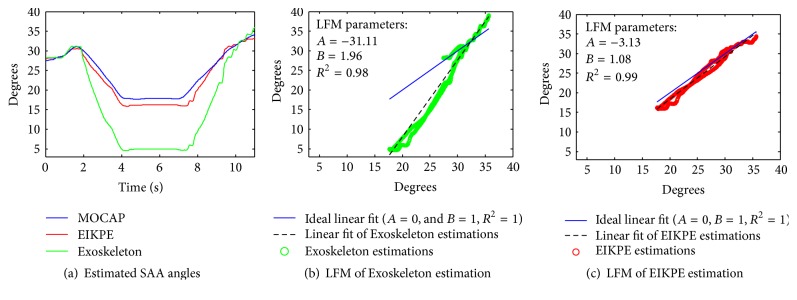
LFM result for the SAA angle estimations of one of the trials of a test subject.

**Figure 9:**
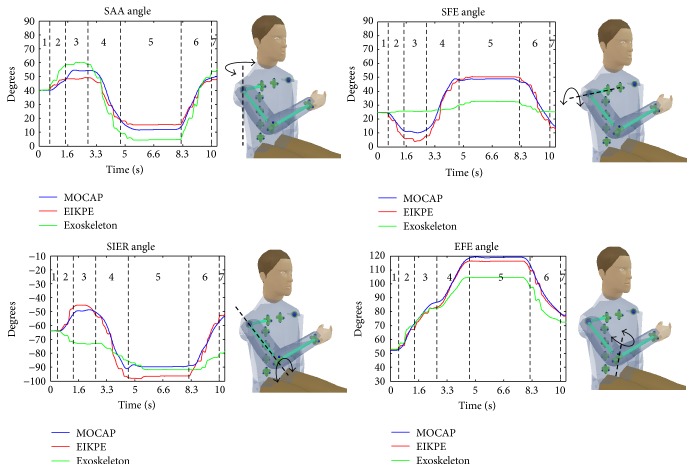
Measurement and estimations of the angles of the shoulder and elbow joints of one of the trials of a test subject. Dashed lines bound the various stages of the prehension movement (Figure 5).

The movement trial in Figure 9 is a good example of the large angle estimation errors produced by the misalignment of the axes of the Exoskeleton joints with respect to the ones of human joints. This misalignment causes under- or overestimation of an angle and also failures in estimating the direction of motion. The SIER is the worst estimated angle by the Exoskeleton (Tables 4 and 5). The low shape similarity coefficient (*R* ^2^ = 0.54) of SIER angle estimations (Table 5) confirms the significant misalignment between the rotation axis of joint 6 of the Exoskeleton (Figure 6) and the upper arm longitudinal axis in most of the ADL stages (Figure 5). According to Table 5, the Exoskeleton tends to overestimate the SAA and to underestimate the SFE.

### 3.2. Angle Estimations of the Elbow Joint

EIKPE presents a fair accuracy in estimating the EFE angle according to the RMSE, ROM, and LFM metrics (Tables 4 and 5). A source of error in the estimation of the EFE angle is in the modeling of the elbow joint. Traditionally, the EFE DOF has been modeled with a revolute joint with its rotation axis normal to both the upper arm and forearm links [35], which is the one used in the EIKPE model. However, the angle between the EFE axis of rotation and the upper arm and forearm longitudinal axes differs between subjects [36] and even varies with the angle of flexion of the elbow [37]. In the case of the MOCAP, the mentioned axis of rotation is estimated by using markers installed on bony landmarks of the elbow at the system calibration stage.

According to Tables 4 and 5, the EFE is the angle that is best estimated by the Exoskeleton (underestimation trend). Notice that the rotation axis of joint 4 of the Exoskeleton is always aligned with the gravitational vertical axis (Figure 6). Then, for the studied ADL, in which the forearm lies on the horizontal plane and reaches the height of the chest, the angle of joint 4 fairly resembles the EFE angle of the subjects. However, it should be remarked that such accuracy will not be maintained when the EFE movement is performed in another plane, as it occurred in the movement trial in Figure 9 (object holding up stage).

### 3.3. Comparison of the Accuracy of the Exoskeleton and EIKPE

For the GH joint angles, Tables 4 and 5 show that EIKPE provides significantly better estimations than the Exoskeleton.

**Figure 10:**
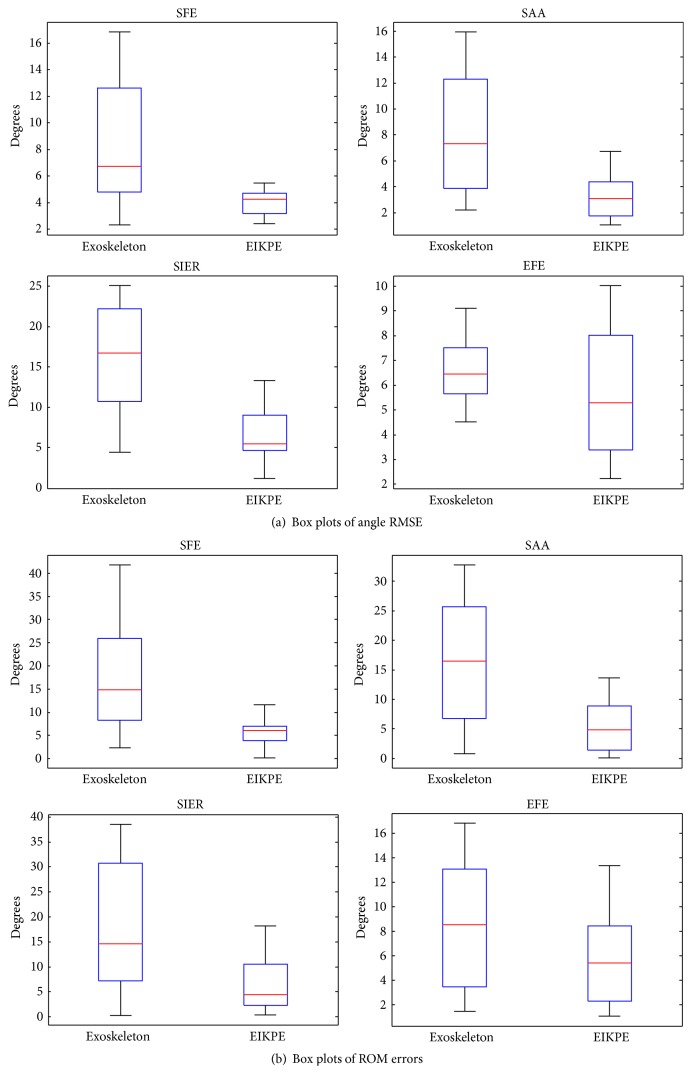
Box plots of (a) RMSE of angle estimations and (b) ROM errors provided by the Exoskeleton and EIKPE for the assessed joint angles.

*(i) RMSE.* EIKPE errors are 50 to 60% lower than those of the Exoskeleton (statistically significant difference).  Figure 10(a) shows that EIKPE variances are significantly lower than those of the Exoskeleton.

*(ii) ROM Error.* EIKPE errors are 60 to 68% lower than those of the Exoskeleton (statistically significant difference).  Figure 10(b) shows that EIKPE variances are significantly lower than those of the Exoskeleton.

*(iii) LFM.* EIKPE parameters are clearly closer to the ideal LFM values than the Exoskeleton ones. EIKPE parameters are consistent across the various angle estimations, in opposition with the Exoskeleton results.

For the EFE angle, Tables 4 and 5 show that EIKPE provides slightly better estimations than the Exoskeleton.

*(i) RMSE.* EIKPE error is 13% lower than that of the Exoskeleton (no statistically significant difference).  Figure 10(a) shows that EIKPE variance is larger than that of the Exoskeleton.

*(ii) ROM Error.* EIKPE error is 35% lower than that of the Exoskeleton (no statistically significant difference).  Figure 10(b) shows that EIKPE variance is lower than that of the Exoskeleton.

*(iii) LFM.* EIKPE parameters are slightly closer to the ideal LFM values than the Exoskeleton ones. The biggest difference between EIKPE and the Exoskeleton is in parameter *B*, meaning that EIKPE estimates better the movement amplitude.

Table 6 shows the global performance metrics for the Exoskeleton and EIKPE angle estimations. Regarding the LFM, this table reports the deviations (mean absolute error (MAE)) of the parameters of the linear fits of the estimations with respect to their ideal values (*A* = 0, *B* = 1, and *R* ^2^ = 1). This table shows that the EIKPE estimation improvements in terms of the LFM parameters are in the order of magnitude of those of the global RMSE and ROM errors (49–60%).

**Figure 11:**
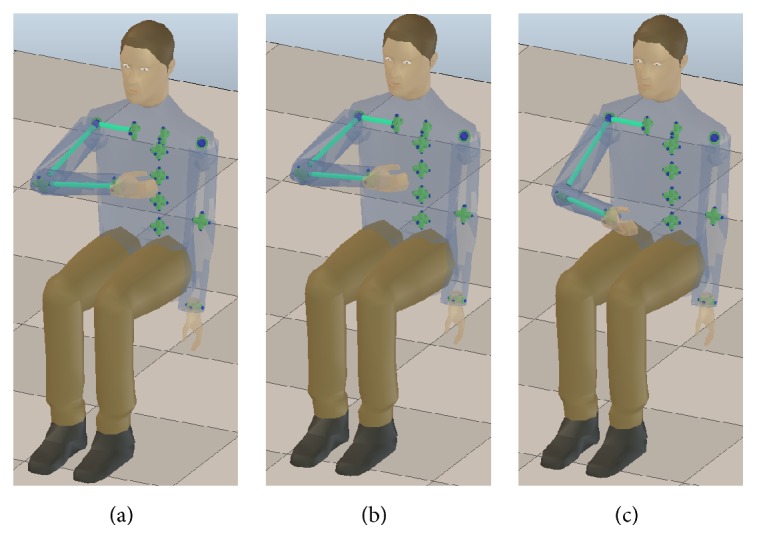
Reconstructed poses of the upper limb at the object holding up stage of the trial depicted in Figure 9 with the joint angle measurements of the (a) MOCAP, (b) EIKPE, and (c) Exoskeleton.

A visual guide of how the joint angle errors are mapped to the reconstructed pose of the upper limb is shown in Figure 11. This figure presents a comparison of the reconstructed upper limb poses at the object holding up stage of the movement trial depicted in Figure 9 with the joint angle estimations provided by the MOCAP, EIKPE, and Exoskeleton.

### 3.4. Comparison with Related Works

The conducted literature review did not produce any other citations than [19–21, 23] in the area of posture estimation of the upper limb in Exoskeleton-based rehabilitation by using computational methods. We consider that the method in [21] would be the strongest competitor to EIKPE (Table 1). Notice that the arm swivel angle representation may suffice for the targeted application in [21]. However, for the application addressed in this work (patient follow-up and evaluation), the joint angles of the limb are required. A direct comparison of EIKPE with the method in [21] is not possible because in this reference only the arm swivel angle is reported.

EIKPE accuracy is close to the ones of MOCAPs that deal with the upper limb posture estimation in ambulatory settings (no robotic devices interacting with the subjects are involved). For instance, the method in [14] presents an average RMSE of 5.5 deg. in the estimation of the angles of the shoulder and elbow joints by using inertial sensors during the ADL movement of reaching for a doorknob.

## 4. Conclusion

This paper studied the feasibility of using the EIKPE method for the estimation of the patient limb posture in the Robot-Assisted Rehabilitation (RAR) of the compound movement of object prehension. In order to do so, the comparison of the estimations of the GH and elbow joint angles provided by (a) EIKPE, (b) the joint encoders of a state-of-the-art commercial Exoskeleton (typical practice in RAR), and (c) an optical motion capture system (ground-truth) was conducted.

The performed test intended to replicate the conditions of use of EIKPE by an end-user. In this way, the estimation of parameters that affect the method accuracy, such as the ones related to the kinematic model of the human subject (arm, forearm, and hand lengths) and to the Exoskeleton kinematic model (adjustable link lengths), was not optimized in any way.

The obtained results suggest that EIKPE is accurate for the application. The studied joint angles were estimated with a RMSE of 5 degrees with respect to the measurements of the optical motion capture system. EIKPE accuracy approaches the one of inertial MOCAPs, avoiding the difficulty of using MOCAPs in RAR.

EIKPE improved markedly the accuracy of the estimations of the GH joint angles provided by the Exoskeleton. Statistically significant differences were found in the accuracy of the Exoskeleton and EIKPE for all GH joint angles. EIKPE provided errors in terms of the LFM parameters, RMS and ROM that are 49–60% smaller than the ones of the Exoskeleton for all the studied angles. This suggests that EIKPE may be used to enhance the accuracy in the estimation of the patient posture in Exoskeleton-based rehabilitation platforms.

*Future Research Opportunities.* The methodology introduced in this paper implies the following future activities for interested researchers: (a) a full clinical study with a patient set (e.g., stroke and spinal cord injury), (b) tests on other Exoskeleton-based platforms, and (c) tests with other compound movements. All of these activities are a natural follow-up given the enhanced posture estimation via the fixture constraints applied here.

## Figures and Tables

**Table 1:** Contributions of this paper with respect to closely related works.

Work	Method	Method inputs	Method evaluation	Metrics
[23]	EIKPE applied to single 1-DOF joint movements	(a) Exoskeleton and Human link lengths (b) Exoskeleton joint angles	(1) Studied angles: elbow and wrist joint angles (2) Reference angles: obtained from marker-based MOCAP (3) Movements: single 1-DOF elbow and wrist joint movements (wearing the Exoskeleton)	RMSE of elbow and wrist angles

[21]	Estimation of the arm swivel angle such that the hand is efficiently retracted towards the head region	(a) Shoulder position (b) Initial elbow position (c) Wrist pose (d) Point on the head region that minimizes the estimation errors	(1) Studied angles: arm swivel angle (2) Reference angles: obtained from redundant marker-based MOCAP (3) Movements: compound movements of (a) object reaching and (b) rotation of a doorknob (not wearing the Exoskeleton)	Mean absolute error of the arm swivel angle

This paper	EIKPE applied to compound movements (multi-DOF and multi-joint)	Same as [23]	(1) Studied angles: GH and elbow joint angles (2) Reference angles: obtained from redundant marker-based MOCAP (3) Movements: compound movement of object prehension (wearing the Exoskeleton)	(i) LFM, RMSE, and ROM error of the GH and elbow angle estimations provided by the Exoskeleton and EIKPE (ii) Statistical significance test of the results of RMSE and ROM errors

**Table 2:** Marker setup for upper limb motion tracking.

Markers on bony landmarks	Markers on body segments
Individual markers are located on the following: (1) Left and right acromion (2) Right iliac crest (3) Lateral and medial epicondyles of the right elbow (4) Radial and ulnar styloid processes of the right wrist (5) Third metacarpal head of the right hand	Marker clusters (groups of 3 markers) are located on the following: (1) Trunk (2) Upper arm (3) Forearm (4) Hand dorsal Only the hand cluster is not rigid

**Table 3:** Method to compute the limb joint angles of interest with the various measuring systems.

Limb joint	Angle	Method to compute the limb joint angles		
		MOCAP	EIKPE	Exoskeleton
GH	SFE	Euler angle decomposition of the rotations of the upper arm marker CS with respect to the thorax marker CS (Figure 3)	Euler angle decomposition of the rotations of the upper arm CS with respect to the Exoskeleton reference CS (Figure 6)	Angle of joint 2 (Figure 6)
	SAA			Sum of the angles of joints 0 and 1 (Figure 6)
	SIER			Angle of joint 6 (Figure 6)

Elbow	EFE	Euler angle decomposition of the rotations of the forearm marker CS with respect to the upper arm marker CS (Figure 3)	Euler angle decomposition of the rotations of the forearm CS with respect to the upper arm CS (Figure 6)	Angle of joint 4 (Figure 6)

**Table 4:** ROM and RMSE metrics (mean ± std. dev.) of the angle estimations provided by the Exoskeleton and EIKPE. Values in bold indicate statistically significant differences in the accuracy of the approaches (*p* value ≤ 0.05).

Metric	Exoskeleton	EIKPE	Improvement^a^
RMSE of SFE angle	**8.4 ± 4.7**	**3.9 ± 0.9**	53%
ROM error of SFE angle	**18.0 ± 11.6**	**5.7 ± 2.8**	68%
RMSE of SAA angle	**8.2 ± 4.6**	**3.3 ± 1.9**	60%
ROM error of SAA angle	**16.9 ± 10.3**	**5.6 ± 4.7**	67%
RMSE of SIER angle	**16.2 ± 6.6**	**6.5 ± 3.1**	60%
ROM error of SIER angle	**17.4 ± 12.6**	**6.9 ± 5.6**	60%
RMSE of EFE angle	6.6 ± 1.4	5.8 ± 2.7	13%
ROM error of EFE angle	8.8 ± 5.5	5.7 ± 3.7	35%

^a^Error reduction with respect to the Exoskeleton by using EIKPE.

**Table 5:** Assessment of estimation methods in terms of the LFM parameters.

Angle	Exoskeleton			EIKPE		
	*A* (deg.)^a^	*B*	*R* ^2^	*A* (deg.)	*B*	*R* ^2^
SFE	25.6 ± 6.7	0.35 ± 0.16	0.75 ± 0.13	−0.53 ± 14.3	0.99 ± 0.36	0.83 ± 0.26
SAA	−22.3 ± 19.2	1.69 ± 0.59	0.91 ± 0.16	−0.87 ± 12.3	1.01 ± 0.32	0.91 ± 0.17
SIER	−17.8 ± 42.9	0.85 ± 0.75	0.54 ± 0.26	11.65 ± 11.9	1.23 ± 0.32	0.85 ± 0.18
EFE	10.6 ± 10.7	0.86 ± 0.15	0.94 ± 0.04	7.18 ± 6.6	0.97 ± 0.18	0.96 ± 0.05

^a^Expected (ideal) values of the LFM parameters are *A* = 0, *B* = 1, and *R* ^2^ = 1.

**Table 6:** Global estimation performance metrics.

Error metric	Exoskeleton	EIKPE	Improvement^a^
Global angle RMSE (deg.)	10.5	5.1	52%
Global ROM error (deg.)	15.3	6.0	60%
MAE in *A* parameter (deg.)	24.5	10.1	59%
MAE in *B* parameter	0.52	0.22	57%
MAE in *R* ^2^ parameter	0.21	0.11	49%

^a^Error reduction with respect to the Exoskeleton by using EIKPE.

## Glossary

ADL: Activity of daily living
CS: Coordinate system
DOF: Degree of freedom
EFE: Elbow flexion-extension
EIKPE: Extended Inverse Kinematics Posture Estimation
GH: Gleno-Humeral
IK: Inverse Kinematics
LFM: Linear fit method
MAE: Mean absolute error
MOCAP: Motion capture system
RAR: Robot-Assisted Rehabilitation
ROM: Range of motion
RMS: Root mean square
RMSE: Root mean square error
SAA: Shoulder abduction-adduction
SFE: Shoulder flexion-extension
SIER: Shoulder internal-external rotation
VR: Virtual reality.

## References

[1] Guidali M., Duschau-Wicke A., Broggi S., Klamroth-Marganska V., Nef T., Riener R. (2011). A robotic system to train activities of daily living in a virtual environment. *Medical & Biological Engineering & Computing*.

[2] Frisoli A., Procopio C., Chisari C. (2012). Positive effects of robotic exoskeleton training of upper limb reaching movements after stroke. *Journal of NeuroEngineering and Rehabilitation*.

[3] Gilliaux M., Lejeune T., Detrembleur C., Sapin J., Dehez B., Stoquart G. (2012). A robotic device as a sensitive quantitative tool to assess upper limb impairments in stroke patients: a preliminary prospective cohort study. *Journal of Rehabilitation Medicine*.

[4] Alankus G., Lazar A., May M., Kelleher C. Towards customizable games for stroke rehabilitation.

[5] Borghese N. A., Murray D., Paraschiv-Ionescu A., Ma M., Jain L. C., Anderson P. (2014). Rehabilitation at home: a comprehensive technological approach. *Virtual, Augmented Reality and Serious Games for Healthcare 1*.

[6] Nordin N., Xie S. Q., Wünsche B. (2014). Assessment of movement quality in robot—Assisted upper limb rehabilitation after stroke: a review. *Journal of NeuroEngineering and Rehabilitation*.

[7] De Los Reyes-Guzmán A., Dimbwadyo-Terrer I., Trincado-Alonso F., Monasterio-Huelin F., Torricelli D., Gil-Agudo A. (2014). Quantitative assessment based on kinematic measures of functional impairments during upper extremity movements: a review. *Clinical Biomechanics*.

[8] Zariffa J., Kapadia N., Kramer J. L. K. (2012). Relationship between clinical assessments of function and measurements from an upper-limb robotic rehabilitation device in cervical spinal cord injury. *IEEE Transactions on Neural Systems and Rehabilitation Engineering*.

[9] Cacho E. W. A., de Oliveira R., Ortolan R. L., Varoto R., Cliquet A. (2011). Upper limb assessment in tetraplegia: clinical, functional and kinematic correlations. *International Journal of Rehabilitation Research*.

[10] Mahoney F. I., Barthel D. W. (1965). Functional evaluation: the barthel index. *Maryland State Medical Journal*.

[11] Keith R. A., Granger C. V., Hamilton B. B., Sherwin F. S. (1987). The functional independence measure: a new tool for rehabilitation. *Advances in Clinical Rehabilitation*.

[12] Zhou H., Hu H. (2008). Human motion tracking for rehabilitation—a survey. *Biomedical Signal Processing and Control*.

[13] Daponte P., De Vito L., Riccio M., Sementa C. Experimental comparison of orientation estimation algorithms in motion tracking for rehabilitation.

[14] El-Gohary M., McNames J. (2012). Shoulder and elbow joint angle tracking with inertial sensors. *IEEE Transactions on Biomedical Engineering*.

[15] Freeman C. T., Rogers E., Hughes A.-M., Burridge J. H., Meadmore K. L. (2012). Iterative learning control in health care: electrical stimulation and robotic-assisted upper-limb stroke rehabilitation. *IEEE Control Systems*.

[16] Riener R., Harders M. (2012). Virtual reality for rehabilitation. *Virtual Reality in Medicine*.

[17] Zhang H., Balasubramanian S., Wei R. RUPERT closed loop control design.

[18] Kousidou S., Tsagarakis N. G., Smith C., Caldwell D. G. Task-orientated biofeedback system for the rehabilitation of the upper limb.

[19] Kim H., Miller L. M., Byl N., Abrams G. M., Rosen J. (2012). Redundancy resolution of the human arm and an upper limb exoskeleton. *IEEE Transactions on Biomedical Engineering*.

[20] Li Z., Kim H., Milutinović D., Rosen J. (2013). Synthesizing redundancy resolution criteria of the human arm posture in reaching movements. *Redundancy in Robot Manipulators and Multi-Robot Systems*.

[21] Kim H., Rosen J. (2015). Predicting redundancy of a 7 DOF upper limb exoskeleton toward improved transparency between human and robot. *Journal of Intelligent & Robotic Systems: Theory and Applications*.

[22] Wang Y., Artemiadis P. (2013). Closed-form inverse kinematic solution for anthropomorphic motion in redundant robot arms. *Advances in Robotics & Automation*.

[23] Cortés C., Ardanza A., Molina-Rueda F. (2014). Upper limb posture estimation in robotic and virtual reality-based rehabilitation. *BioMed Research International*.

[24] Denavit J., Hartenberg R. S. (1955). A kinematic notation for lower-pair mechanisms based on matrices. *Transactions of the ASME*.

[25] Hocoma A. G. http://www.hocoma.com/products/armeo/armeospring/.

[26] Coppelia Robotics http://www.coppeliarobotics.com/.

[27] de Los Reyes-Guzmán A., Gil-Agudo A., Peñasco-Martín B., Solís-Mozos M., del Ama-Espinosa A., Pérez-Rizo E. (2010). Kinematic analysis of the daily activity of drinking from a glass in a population with cervical spinal cord injury. *Journal of NeuroEngineering and Rehabilitation*.

[28] http://www.c-motion.com/products/visual3d/.

[29] Charnwood Dynamics http://www.codamotion.com/index.php/applications/hardware.

[30] Yeong C. F., Melendez-Calderon A., Burdet E. Analysis of pick-and-place, eating and drinking movements for the workspace definition of simple robotic devices.

[31] Iosa M., Cereatti A., Merlo A., Campanini I., Paolucci S., Cappozzo A. (2014). Assessment of waveform similarity in clinical gait data: the linear fit method. *BioMed Research International*.

[32] Castelli A., Paolini G., Cereatti A., Croce U. D. (2015). A 2D markerless gait analysis methodology: validation on healthy subjects. *Computational and Mathematical Methods in Medicine*.

[33] Woolson R. F. (2008). *Wilcoxon Signed-Rank Test*.

[34] http://www-01.ibm.com/software/analytics/spss/.

[35] Laitenberger M., Raison M., Périé D., Begon M. (2015). Refinement of the upper limb joint kinematics and dynamics using a subject-specific closed-loop forearm model. *Multibody System Dynamics*.

[36] Paraskevas G., Papadopoulos A., Papaziogas B., Spanidou S., Argiriadou H., Gigis J. (2004). Study of the carrying angle of the human elbow joint in full extension: a morphometric analysis. *Surgical and Radiologic Anatomy*.

[37] Van Roy P., Baeyens J.-P., Fauvart D., Lanssiers R., Clarijs J. P. (2005). Arthro-kinematics of the elbow: study of the carrying angle. *Ergonomics*.

